# Efficacy of bimatoprost for the treatment of primary open-angle glaucoma: A protocol of systematic review and meta-analysis

**DOI:** 10.1097/MD.0000000000020356

**Published:** 2020-06-05

**Authors:** Hong-wei Liu, Yu-tong Lu, Yong-bo Ren, Yan Meng

**Affiliations:** Department of Ophthalmology, First Affiliated Hospital of Jiamusi University, Jiamusi, China.

**Keywords:** bimatoprost, efficacy, primary open-angle glaucoma, safety

## Abstract

**Background:**

Bimatoprost has been reported to treat primary open-angle glaucoma (POAG) effectively. However, up-to-date, no systematic review has specifically addressed the efficacy and safety of bimatoprost for the treatment of POAG. Therefore, this study will propose to appraise the efficacy and safety of bimatoprost for the treatment of POAG.

**Methods:**

We will perform a systematic search in MEDLINE, EMBASE, CINAHI, Cumulative Index to Nursing and Allied Health Literature, Allied and Complementary Medicine Database, Web of Science, Cochrane Library, Chinese Biomedical Literature Database, and China National Knowledge Infrastructure from inception up to the March 1, 2020. We will include randomized controlled trials (RCTs) for evaluating the efficacy and safety of bimatoprost for the treatment of POAG. Primary outcome is the mean intraocular pressure (IOP) reduction from baseline to the endpoint, and change in best corrected visual acuity. Secondary outcomes are contrast sensitivity, rate of progression of glaucoma, quality of life, and incidence of adverse events. Study quality will be examined by Cochrane Collaboration tool, and strength of evidence will be evaluated by Grading of Recommendations Assessment Development and Evaluation tool.

**Results:**

This proposed study will outline the current RCTs to assess the efficacy and safety of bimatoprost for the treatment of POAG.

**Conclusion:**

The findings of this study will confirm whether bimatoprost is beneficial to patients with POAG.

**Systematic review registration:**

INPLASY202040118.

## 1 Introduction

Glaucoma is a very common eye disorder, which leads to the visual impairment and irreversible blindness.^[1–3]^ Its prevalence rate was 3.54% with estimated 64.3 million patients in 2013.^[4,5]^ This figure is estimated to increase up to 18.2% in 2020 and 73.8% in 2040.^[4,5]^ If this condition can not be managed effectively, it may significantly affect quality of life in such patients.^[6,7]^ Thus, effective treatments are very important to preserve visual function, and to improve quality of life.

Bimatoprost has been used to treat POAG in the past few years,^[8–18]^ and several studies have found promising efficacy of bimatoprost for the treatment of POAG.^[9–18]^ However, no evidence from systematic review has been provided. Thus, this study will collect evidence of the efficacy and safety of bimatoprost in the treatment of POAG to determine whether it can benefit patients with POAG.

## 2 Methods and analysis

### 2.1 Study registration

This protocol of this study has been registered on INPLASY202040118, and has organized following the Preferred Reporting Items for Systematic Reviews and Meta-analysis Protocol.^[19]^

### 2.2 Study selection criteria

#### 2.2.1 Types of studies

Only consider randomized controlled trials (RCTs) will be qualified in this research. The literatures of animal studies, comments, reviews, case reports, case series, non-RCTs, uncontrolled trials, and quasi-RCTs will not be included.

#### 2.2.2 Types of participants

The research patients were definitely diagnosed as POAG, and there will be no restrictions related to the country, age, sex, and other relevant factors.

#### 2.2.3 Types of interventions

Studies implemented bimatoprost alone as an experimental treatment regardless its delivery methods, duration, dosage, and frequency.

Apart from bimatoprost, there are no restrictions to the control interventions.

#### 2.2.4 Types of outcomes

##### 2.2.4.1 Primary outcome

1. Mean intraocular pressure reduction from baseline to the endpoint;
2. Change in best corrected visual acuity.

##### 2.2.4.2 Secondary outcome

1. Contrast sensitivity;
2. Rate of progression of glaucoma;
3. Quality of life;
4. Incidence of adverse events.

### 2.3 Search strategy for study identification

#### 2.3.1 Electronic databases searches

The below electronic database resources will be searched from inception up to the March 1, 2020: MEDLINE, EMBASE, CINAHI, Cumulative Index to Nursing and Allied Health Literature, Allied and Complementary Medicine Database, Web of Science, Cochrane Library, Chinese Biomedical Literature Database, and China National Knowledge Infrastructure. We will include RCTs for assessing the efficacy and safety of bimatoprost for the treatment of POAG. The detailed search strategy of Cochrane Library is placed in Table 1. Similar search strategy with specifics for other electronic databases will be presented.

**Table 1:**
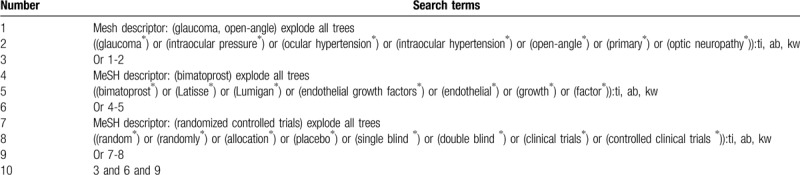
Search strategy of Cochrane Library.

#### 2.3.2 Other resources searches

Clinical trials registry, conference/meeting proceedings and reference lists of relevant reviews will be examined to avoid omission.

### 2.4 Study selection

**Figure 1:**
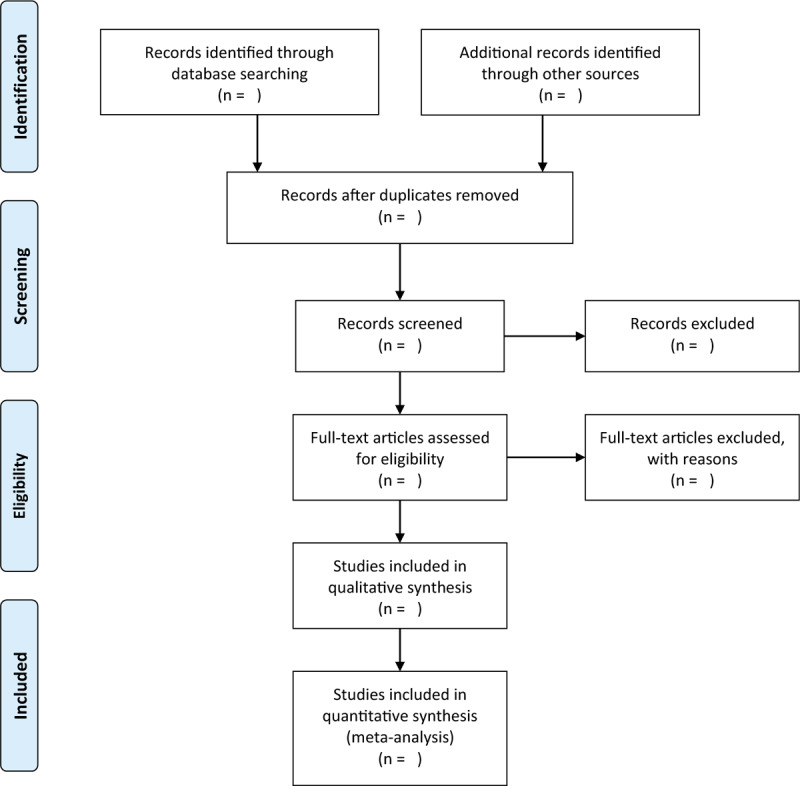
Process of study selection.

EndNote X9 software will be applied to manage all retrieved records, and all duplicates will be removed. Then, 2 researchers will independent scan selected records according to titles and abstracts. All irrelevant articles will be eliminated. After the preliminary evaluation, we will examine the full-text of potential trials based on the eligibility criteria. Regarding the divergences arising between researchers, a third researcher will help to solve them by discussion. We will utilize a flow diagram to summarize the process of study selection (Fig. 1).

### 2.5 Data extraction and management

Two researchers will independently extract data using previous designed standard data extraction sheet. Disagreements regarding data extraction will be settled by consulting a third researcher. The content includes title, first author, year of publication, country, patient characteristics, study design, trial setting, interventions, comparators, outcomes, results, findings, follow-up data, conflict of interests, and other associated information.

### 2.6 Missing data dealing with

If we identify unclear or missing data, we will contact original authors to request it. We will analyze available data if we can not obtain that data.

### 2.7 Risk of bias assessment

Two researchers will independently appraise the risk of bias for each eligible trial based on 7 items using The Cochrane Handbook for Systematic Reviews of Interventions Tool.^[20]^ Each item is rated as high, unclear, or low risk of bias. Confusion in the interpretation will be solved by a third researcher through discussion.

### 2.8 Appraising quality of evidence

Two researchers will independently appraise overall strength of the evidence using Grading of Recommendations Assessment, Development and Evaluation tool.^[21]^ We will summarize its results in tables of Summary of Findings. Differences will be figured out through consultation with the help of a third researcher.

### 2.9 Statistical analysis

All data will be analyzed using RevMan 5.3 software. All continuous variables will be calculated as mean difference or standardized mean difference and 95% confidence intervals (CIs). All dichotomous variables will be estimated as risk ratio and 95% CIs. Chi-Squared test and *I*^2^ statistic will be applied to examine the heterogeneity of eligible trials. *P* > .1 and/or *I*^2^ < 50% suggests acceptable heterogeneity, and we will use a fixed-effects model; while *P* ≤ 0.1 and/or *I*^2^ ≥ 50% indicates obvious significant heterogeneity, and we will utilize a random-effects model. If acceptable heterogeneity is examined and sufficient data are collected, we will carry out a meta-analysis according to the similarity in study characteristics, interventions, controls, and outcomes. If obvious heterogeneity is tested, we will perform a subgroup analysis to explore the sources of heterogeneity according to the variations in intervention types, research scenario, and outcome tools. In addition, we will also place a sensitivity analysis to test the robustness of study findings by removing trials with high risk of bias. Whenever possible, we will also conduct a funnel plot and Egger regression test to check reporting bias if over 10 trials are included.

### 2.10 Dissemination and ethics

2.10

The results of this study are expected to be published on a peer-reviewed journal or relevant conference. It is a literature-based study; therefore it does not require ethical approval.

## 3 Discussion

Recent studies reported that bimatoprost has been utilized for the treatment of POAG.^[8–18]^ However, whether bimatoprost can benefit and play an ideal role in the treatment of POAG is still unclear at evidence-based medicine level. As far as we know, this study is the first one to investigate the efficacy and safety of bimatoprost for the treatment of POAG, so this study can fill the gap. The results of this study will present evidence to judge whether bimatoprost is effective and safety for the treatment of POAG.

## Author contributions

**Conceptualization:** Hong-wei Liu, Yong-bo Ren.

**Data curation:** Hong-wei Liu, Yu-tong Lu, Yan Meng.

**Formal analysis:** Hong-wei Liu, Yong-bo Ren, Yan Meng.

**Funding acquisition:** Yan Meng.

**Investigation:** Yan Meng.

**Methodology:** Hong-wei Liu, Yu-tong Lu, Yong-bo Ren.

**Project administration:** Yan Meng.

**Resources:** Hong-wei Liu, Yu-tong Lu.

**Software:** Hong-wei Liu, Yu-tong Lu, Yong-bo Ren.

**Supervision:** Yan Meng.

**Validation:** Hong-wei Liu, Yu-tong Lu, Yong-bo Ren, Yan Meng.

**Visualization:** Hong-wei Liu, Yan Meng.

**Writing – original draft:** Hong-wei Liu, Yu-tong Lu, Yong-bo Ren, Yan Meng.

**Writing – review & editing:** Hong-wei Liu, Yu-tong Lu, Yan Meng.
